# Inhibitor-induced oxidation of the nucleus and cytosol in *Arabidopsis thaliana:* implications for organelle to nucleus retrograde signalling

**DOI:** 10.1098/rstb.2016.0392

**Published:** 2017-08-14

**Authors:** Barbara Karpinska, Sarah Owdah Alomrani, Christine H. Foyer

**Affiliations:** Centre for Plant Sciences, School of Biology, Faculty of Biological Sciences, University of Leeds, Leeds LS2 9JT, UK

**Keywords:** antimycin A, alternative oxidase, lincomycin, nuclear-encoded mitochondrial proteins, norflurazon, photosynthesis-associated nuclear genes

## Abstract

Concepts of organelle-to-nucleus signalling pathways are largely based on genetic screens involving inhibitors of chloroplast and mitochondrial functions such as norflurazon, lincomycin (LINC), antimycin A (ANT) and salicylhydroxamic acid. These inhibitors favour enhanced cellular oxidation, but their precise effects on the cellular redox state are unknown. Using the *in vivo* reduction–oxidation (redox) reporter, roGFP2, inhibitor-induced changes in the glutathione redox potentials of the nuclei and cytosol were measured in *Arabidopsis thaliana* root, epidermal and stomatal guard cells, together with the expression of nuclear-encoded chloroplast and mitochondrial marker genes. All the chloroplast and mitochondrial inhibitors increased the degree of oxidation in the nuclei and cytosol. However, inhibitor-induced oxidation was less marked in stomatal guard cells than in epidermal or root cells. Moreover, LINC and ANT caused a greater oxidation of guard cell nuclei than the cytosol. Chloroplast and mitochondrial inhibitors significantly decreased the abundance of *LHCA1* and *LHCB1* transcripts. The levels of *WHY1*, *WHY3* and *LEA5* transcripts were increased in the presence of inhibitors. Chloroplast inhibitors decreased *AOXA1* mRNA levels, while mitochondrial inhibitors had the opposite effect. Inhibitors that are used to characterize retrograde signalling pathways therefore have similar general effects on cellular redox state and gene expression.

This article is part of the themed issue ‘Enhancing photosynthesis in crop plants: targets for improvement’.

## 1. Introduction

Organelle-to-nucleus retrograde signalling pathways coordinate nuclear gene expression with chloroplast and mitochondrial functions. Respiratory and photosynthetic electron transport and energy metabolism are performed by multi-subunit complexes that are composed of proteins encoded by genes housed in the nuclear, mitochondrial or plastid genomes*.* Precise coordination of the expression of genes encoded by the genomes in the different intracellular compartments is required to facilitate the assembly of functional mitochondria and chloroplasts under fluctuating environmental or metabolic conditions [1–3]. In higher plants, energy metabolism and metabolite trafficking are also coordinated between the mitochondria and chloroplasts to optimize key metabolic pathways such as primary nitrogen assimilation and sucrose synthesis [4,5].

Much of our current understanding of chloroplast-to-nucleus signalling pathways comes from the characterization of *Arabidopsis thaliana genomes uncoupled* (*gun*) mutants [6]. These mutants were isolated using a screen involving exposure to the phytoene desaturase inhibitor, norflurazon (NF). NF-dependent inhibition of carotenoid biosynthesis results in decreased expression of nuclear genes encoding chloroplast proteins, such as the photosynthetic light-harvesting antenna proteins [6], for example *LIGHT-HARVESTING CHLOROPHYLL A/B BINDING PROTEIN B1* (*LHCB1*). Such screens also often use the plastid protein synthesis inhibitor lincomycin (LINC), which blocks chloroplast and mitochondrial protein synthesis but does not affect cytosolic translation. However, the mechanisms by which NF-mediated inhibition of electron transport functions is signalled to the nucleus leading to decreased transcription of photosynthesis-related genes remains poorly understood. Five *genomes uncoupled (gun)* mutants (*gun1–gun5*) have been characterized to date in *A. thaliana* [2,6]. Four of the *GUN* genes (*GUN2–GUN5*) have roles in tetrapyrrole metabolism, *GUN2–GUN5* encoding a haem oxygenase, a phytochromobilin synthase, an Mg-chelatase cofactor and an H-subunit of Mg-chelatase, respectively [2]. Regulation of nuclear gene expression via perturbations in tetrapyrrole metabolism also involves heat shock protein 90 (HSP90) and LONG HYPOCOTYL5, a bZIP transcription factor [7]. The role of the tetrapyrrole pathway intermediate and chlorophyll precursor Mg-protoporphyrin IX in the signalling pathways that regulate nuclear gene expression remains somewhat uncertain [8–11]. However, the activity of the Mg-ProtoIX interacting protein, phytochrome-associated protein phosphatase 5 (PAPP5) is required for chloroplast-to-nucleus retrograde signal transduction, possibly by sensing Mg-ProtoIX accumulation [12].

*GUN1* encodes a chloroplast-localized pentatricopeptide repeat protein [13,14] that requires both a chloroplast envelope-bound PHD transcription factor [15] and the nuclear transcription factor ABA-INSENSITIVE4 (ABI4) to regulate nuclear gene expression [16,17]. The ABI4 transcription factor is also a regulator of the expression of the alternative oxidase (*AOX*); [18], which is a nuclear-encoded mitochondrial gene that has been extensively characterized in terms of mitochondria to nucleus retrograde regulation [3]. Antimycin A (ANT) blocks the cytochrome *c* oxidase-dependent mitochondrial electron transport pathway, leading to the expression of *AOX1a,* which is regarded as a classical mitochondrial stress marker gene that is expressed when mitochondrial energy status is impaired [19]. The AOX pathway functions in energy dissipation, a process that potentially also has benefits for photosynthesis [4]. As signals from the photosynthetic electron transport chain also play a role in regulating the abundance of AOX, it has been suggested that the ABI4 transcription factor might function as a common molecular link in chloroplast-to-nucleus and mitochondria-to-nucleus signalling pathways, facilitating coordinated expression of photosynthesis-associated nuclear genes (PhANGs), such as *LHCB1* and nuclear-encoded mitochondrial proteins (NGEMPs) such as AOX1a [20]. The ABI4 transcription factor is also important in transmission of redox signals that regulate plant growth and defence responses [21–23].

Inhibitors such as LINC, NF and ANT that are commonly used to characterize retrograde signalling, cause an accumulation of reactive oxygen species (ROS), perturbing cellular redox homeostasis and activating oxidative signalling pathways [24,25]. However, the precise effects of these inhibitors on the redox state of the cytosol and nuclei have not been characterized in detail. In the following studies, we therefore examined the effects of LINC, NF, LINC + NF or ANT on the degree of oxidation of the nuclei and cytosol of *A. thaliana* root, epidermal and cotyledon stomatal guard cells using an *in vivo* redox-sensitive fluorescent protein (roGFP2) probe [26], together with the expression of selected nuclear genes that are commonly used as marker PhANGs and NGEMPs.

## 2. Material and methods

Seeds of *A. thaliana* (L.) ecotype Columbia-0 that constitutively express roGFP2 [26] were grown in the absence or the presence of inhibitors using standard protocols [16,21]. Seedlings were grown for 5 days on vertical agar plates containing half strength Murashige and Skoog media containing 0.1 g l^−1^ myoinositol, 10 g l^−1^ sucrose and 0.5 g l^−1^ 2-(*N*-morpholino) ethanesulfonic acid (MES) buffer (pH 5.7). For inhibitor treatments, this medium was supplemented with either LINC (500 µM), NF (5 µM), LINC (500 µM) + NF (5 µM), ANT (20 µM), salicylhydroxamic acid (SHAM) (20 µM, or 5 µM), or ANT (20 µM) + SHAM (20 µM). Each experiment was repeated at least three times and involved 50 seeds per line.

### (a) Confocal microscopy

Seedlings were placed on a slide in a drop of sterile water. Fluorescence imaging was performed using a confocal microscope (Carl Zeiss LSM880, Jena, Germany). The microscope was equipped with 405 and 488 nm lasers for detection of the oxidized and reduced forms of ro-GFP2, respectively. Images were taken with a 40×/1.3 oil DIC M27 lens (Zeiss Objective C-Apochromat 40 × /1.2 W Corr M27) in multi-track mode with line switching between 488 and 405 nm excitation. Ratiometric analyses were performed using ImageJ software (http://rsbweb.nih.gov/ij/). The range of the roGFP2 signal was calibrated at the end of each experiment using 2.5 mM dithiothreitol (DTT, reduced) or 2 mM hydrogen peroxide (oxidized). The oxidation degree and glutathione redox potential values were calculated as described previously [26].

### (b) Quantitative PCR

Real time (qPCR) was performed on total RNA extracted from whole seedlings, essentially as described previously [27] using an Eppendorf Realplex2 real-time PCR system. One-step RT-PCR using a Quantifast SYBR Green RT-PCR Kit (Qiagen) was performed according to the manufacturer's instructions. The expression of the genes of interest using the primer sequences given in table 1 was normalized using *A. thaliana UBIQUITIN 10* as an endogenous control. Each experiment, which involved 10 seedlings per line, was repeated at least three times.

**Table 1.** Accession numbers and primers.

gene no.	annotation	forward primer (5′ → 3′)	reverse primer (5′ → 3′)
AT4G05320	*UBIQUITIN 10* (*UBQ10*)	AGGTATTCCTCCGGACCAGCAG	AGAAACCACCACGAAGACGCAG
AT3G54890	*PHOTOSYSTEM I LIGHT-HARVESTING COMPLEX GENE 1* (*LHCA1*)	AAGCCGACTGTTGCACACAGA	TTGGCCATTGAGTTCTTAGCCA
AT1G29920	*CHLOROPHYLL A/B-BINDING PROTEIN 2* (*LHCB1.1*)	GGCTCTCTCCTCCCCTGCTTTC	CCCTTTGGCTTGGCAACAGTCT
AT1G14410	*WHIRLY1* (*AtWHY1*)	CTGGTGCTCTTGGGTCCACTGT	TTCGAGAAGCAGAGGTTCG
AT2G02740	*WHIRLY3* (*AtWHY3*)	AGCCACAAACTCTGGTGCTCG	CGTCTTCTTCGCAAAACGCTG
AT4G02380	*LATE EMBRYOGENESIS ABUNDANT PROTEIN 5* (*AtLEA5*)	GGGTTCCAGATCCCAAAACC	AGCTCAGCCGCGTCAATCTC
AT3G22370	*ALTERNATIVE OXIDASE 1A* (*AtAOX1A*)	GAGCCTCGTCAGCACGAACAAC	TTGAGAATGTTCCTGCTCCGGC
AT4G34410	*REDOX RESPONSIVE TRANSCRIPTION FACTOR 1* (*RRTF1*)	ATATTGCAATCCCCTCCTCC	GGGCTAAACTCAACTTCCC

## 3. Results

**Figure 1.**
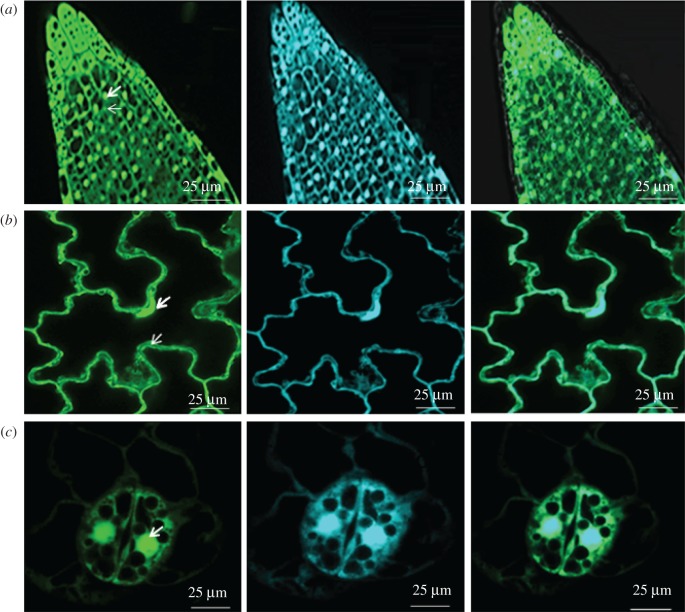
Typical examples of ro-GFP2 fluorescence images of the root and cotyledons of *A. thaliana* seedlings in the absence of inhibitors. Root tip (*a*), leaf epidermal cells (*b*) and stomata guard cell (*c*). In each case, the reduced form (left panels) was determined following incubation with 5 mM DTT, and the oxidized form was determined following incubation with 2 mM hydrogen peroxide (middle panels). The overlaid images of reduced and oxidised forms (right panels) are shown for completeness. Wide arrows indicate nuclei and thin arrows indicate cytosol. Scale bar, 25 µm.

There was no significant difference in seed germination on media in the absence or presence of inhibitors, but the presence of inhibitors had a negative impact on the primary root length of 5-day-old seedlings (data not shown). The control (CTR) seedlings had a mean root length of 0.8 cm. However, the roots of 5-day-old seedlings grown in the presence of NF, LINC, LINC + NF or ANT had a mean length between 0.4 and 0.5 cm (data not shown). Examples of the images of the primary root cells and the epidermal and stomatal guard cells in the cotyledons of the 5-day-old seedlings that were used to determine the effects of the different inhibitors on the redox sate of the nuclei and cytosol are shown in figure 1.

**Figure 2.**
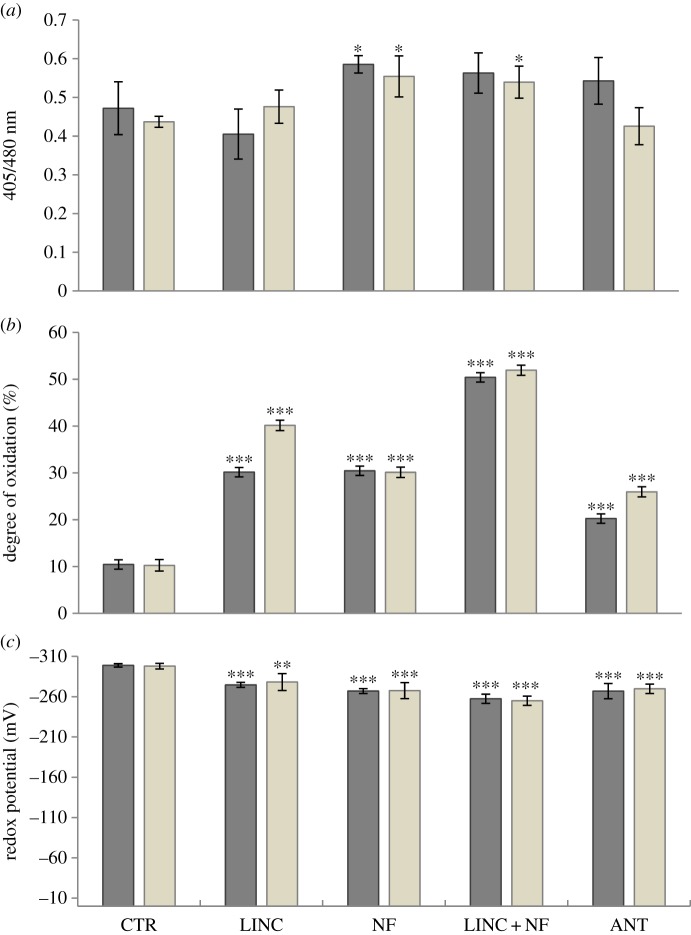
The 405/488 nm fluorescence ratios (*a*), the degree of oxidation (*b*) and the glutathione redox potential (*c*) measured in the cytosol (dark bar) and in the nuclei (grey bar) of the stomatal cells of seedlings grown for 5 days either in the absence (CTR) or the presence of LINC, NF, LINC + NF or ANT. Data are the mean ± s.d. Asterisks indicate the statistical significance of *p*-value: *p ≤ 0.05, **p < 0.01, ***p < 0.001.

Growth in the presence of NF, LINC, LINC + NF or ANT led to changes in the 405/488 nm fluorescence ratio (figure 2*a*), and an increase in the degree of oxidation of the nuclei and cytosol of the stomatal guard cells in the cotyledons (figure 2*b*). The highest overall increase in oxidation was observed in the LINC + NF treatment (about 50% compared to less about 10% in controls), where the nuclei and cytosol were oxidized to similar levels relative to controls (figure 2*b*). The NF and LINC treatments caused less oxidation when these inhibitors were added alone rather than in combination, the application of LINC + NF having an additive effect in terms of the observed increase in oxidation of the nuclei and cytosol of the stomatal guard cells (figure 2*b*). While ANT treatment increased the degree of oxidation, this increase was less than that caused by the NF or LINC treatments. The LINC and ANT treatments led to a significantly greater oxidation of the nuclei than the cytosol (figure 2*b*). All the inhibitors decreased the glutathione redox potentials of the nuclei and cytosol of the stomatal cells (figure 2*c*), the decrease being greatest in the seedlings grown with LINC + NF, which showed a change of about −40 mV.

**Figure 3.**
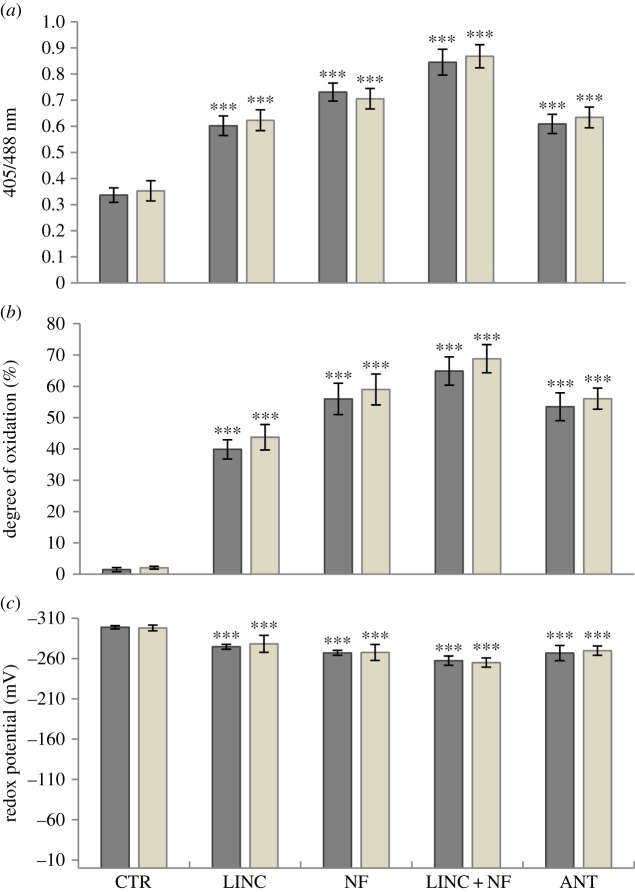
The 405/488 nm fluorescence ratios (*a*), the degree of oxidation (*b*) and the glutathione redox potential (*c*) measured in the cytosol (dark bar) and in the nuclei (grey bar) of the epidermal cells of the cotyledons of seedlings grown for 5 days either in the absence (CTR) or presence of LINC, NF, LINC + NF or ANT. Data are the mean ± s.d. For statistical significance of *p*-values, see figure 2.

Growth in the presence of NF, LINC, LINC + NF and ANT led to changes in the 405/488 nm fluorescence ratios measured in the epidermal cells of the cotyledons (figure 3*a*). In the epidermal cells, however, the degree of oxidation was increased to a similar level in the nuclei and cytosol in the presence of each inhibitor (figure 3*b*). The inhibitors caused a greater increase in the degree of oxidation of the cytosol and nuclei of the epidermal cells of the cotyledons (figure 3*b*) than that observed in the stomatal guard cells (figure 2*b*). The highest overall inhibitor-mediated increase in oxidation (about 70% compared to less than 5% in controls) was observed in the presence of LINC + NF treatment (figure 3*b*). However, the NF and ANT treatments also caused a high level of oxidation (figure 3*b*). The glutathione redox potentials of the nuclei and cytosol of the epidermal cells in the absence of inhibitors was over −300 mV (figure 3*c*). By contrast, the glutathione redox potential of the epidermal cells of seedlings grown in the presence of LINC + NF was about −250 mV. The glutathione redox potentials of the nuclei and cytosol of the epidermal cells maintained slightly higher values in the seedlings grown with ANT, or LINC or NF alone (figure 3*c*).

**Figure 4.**
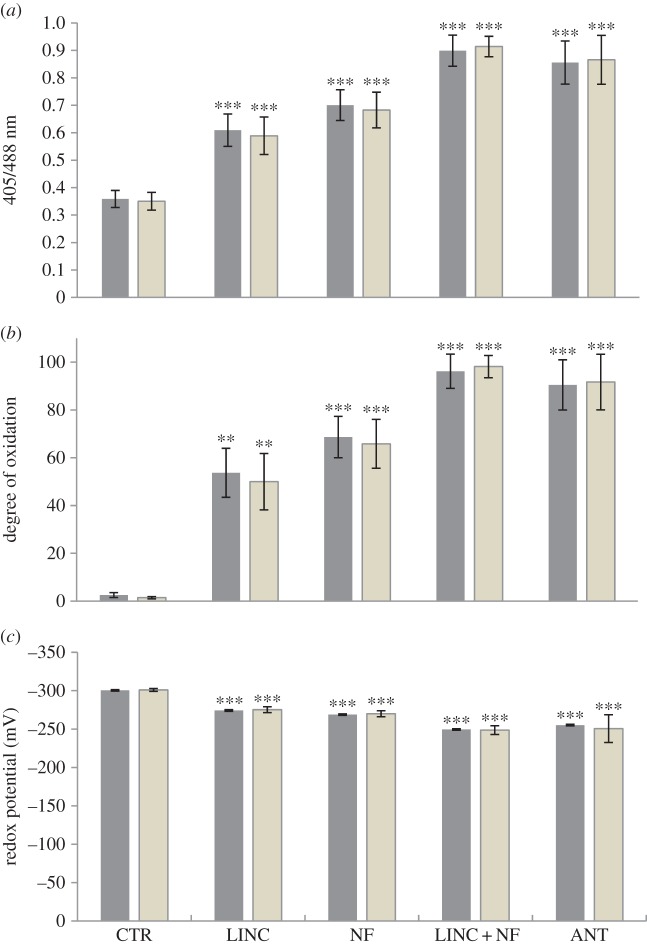
The 405/488 nm fluorescence ratios (*a*), the degree of oxidation (*b*) and the glutathione redox potential (*c*) measured in the cytosol (dark bar) and in the nuclei (grey bar) of the root tips of seedlings grown for 5 days either in the absence (CTR) or the presence of LINC, NF, LINC + NF or ANT. Data are the mean ± s.d. For statistical significance of *p*-values, see figure 2.

The 405/488 nm fluorescence ratios of the proliferating cells in the root apical meristem were also changed as a result of growth in the presence of NF, LINC, LINC + NF or ANT (figure 4*a*). The degree of oxidation was increased to a similar level in the nuclei and cytosol of the proliferating root cells in the presence of each inhibitor (figure 4*b*). Root cells treated with LINC + NF showed the highest degree of oxidation, almost 100% (figure 4*b*). Similarly, the cytosol and nuclei of the ANT-treated roots showed an oxidation degree of about 90% compared to controls, which had values of less than 5%. The roots that had been exposed to NF or LINC alone had much lower levels of oxidation (50–60%) compared to the combined LINC + NF treatment (figure 4*b*). The glutathione redox potentials (figure 4*c*) of the cytosol and nuclei of the root cells in the absence of inhibitors were about −300 mV. The glutathione redox potentials were less changed in the presence of LINC (−270 mV) or NF (260 mV) alone compared to the LINC + NF treatment (figure 4*c*).

**Figure 5.**
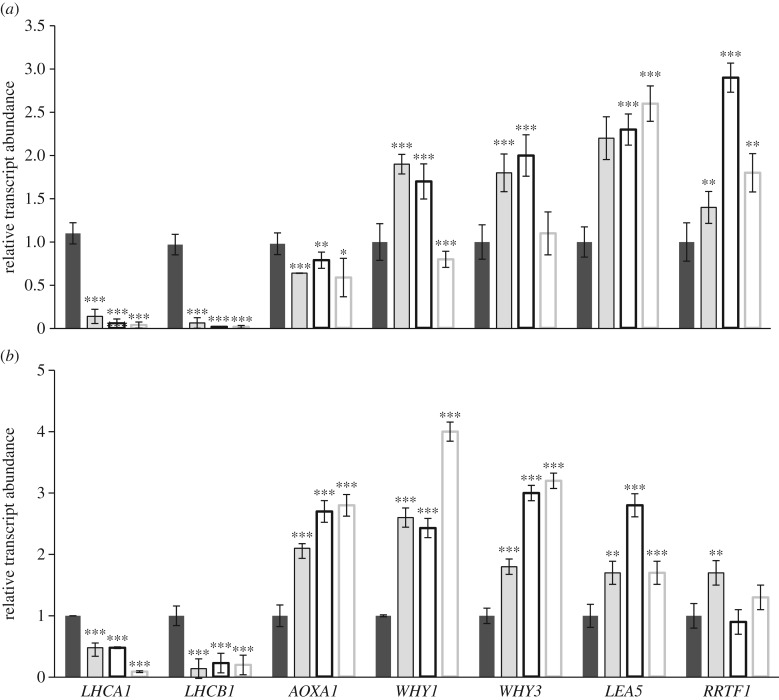
The relative abundance of transcripts in 5-day-old seedlings grown for 5 days either in the absence or presence of inhibitors of chloroplast (*a*) or mitochondrial (*b*) functions. In (*a*), seedlings were either grown in the absence of inhibitors (black bars) or in the presence of LINC (grey bars), NF (white bar with black border) or LINC + NF (white bars with grey border), In (*b*), seedlings were either grown in the absence of inhibitors (black bars) or in the presence of ANT (grey bars), SHAM (white bar with black border), or SHAM plus ANT (white bar with grey border). Data are expressed relative to *UBIQUITIN 10*. Data are the mean ± s.d. For statistical significance of *p*-values, see figure 2. (*a*) *LHCA1, LIGHT-HARVESTING CHLOROPHYLL A BINDING PROTEN 1; LHCB1*, *LIGHT-HARVESTING CHLOROPHYLL A BINDING B1*; *AOXA1, ALTERNATIVE OXIDASE A1*; (*b*) *WHIRLY1* (*WHY1, WHIRLY1; WHY3, WHIRLY3; LEA5,* *LATE EMBRIOGENESIS 5*; *RRTF1*, *REDOX-REGULATED TRANSCRIPTION FACTOR 1*.

The LINC, NF and LINC + NF treatments significantly decreased the abundance of *LHCA1* and *LHCB1* transcripts (figure 5*a*). These inhibitors also decreased the levels of *AOXA1* transcripts, but to a lesser extent than the inhibition of the PhANGs (figure 5*a*). The presence of either LINC or NF, but not LINC + NF, significantly increased the abundance of *WHY1* and *WHY3* transcripts, whereas the levels of *LEA5,* and *RRTF1 mRNAs* were increased by all three treatments (figure 5*a*). These nuclear genes are not generally classed as either PhANGs or NGEMPs.

The treatments with ANT and the AOX inhibitor SHAM significantly decreased the levels of *LHCA1* and *LHCB1* transcripts (figure 5*b*). By contrast, the mitochondrial electron transport inhibitors significantly increased the abundance of *AOXA1, WHY1* and *WHY3* transcripts (figure 5*b*). The levels of *LEA5* mRNAs were also significantly increased in the presence of ANT, SHAM or ANT + SHAM, while the abundance of *RRTF1* mRNAs was only increased by the ANT treatment (figure 5*b*).

## 4. Discussion

Chloroplast and mitochondrial functions depend on the coordinated expression of both organelle and nuclear genomes. Mechanistic insights concerning the complex but poorly understood communication and signalling pathways between the genomes in the plastids, mitochondria and nuclei have largely come from studies using inhibitors such as NF, LINC and ANT. The effects of these inhibitors on the redox state of plant cells were estimated *in vivo* in this study by using a probe that measures the redox potential of glutathione. Such fluorescent ro-GFP proteins, which contain thiol groups that are sensitive to oxidation, have been widely used to monitor the redox status of plant cells [26,28,29]. The probe used in this study has a midpoint potential between −280 and −290 mV and is therefore almost fully reduced in the cytosol, plastids and mitochondria [29]. While this probe is less effective in accurately measuring the glutathione redox potentials occurring under strongly oxidizing conditions [29], the present analysis nevertheless provides useful new data concerning inhibitor-induced oxidation in different cell types.

The data presented here show that 5 days exposure to NF, LINC, LINC + NF or ANT greatly increased the oxidation state of the nuclei and cytosol in both the shoot and root cells. Moreover, the effects of the inhibitors on the degree of oxidation were found to be cell type-specific. In contrast with predictions, the inhibitors appeared to cause less oxidation in the stomatal guard cells than the epidermal or root cells. While it might be possible that the guard cells are less permeable to these inhibitors than the other cell types, the observed differences might also be due to the presence of chloroplasts in the guard cells. In the absence of inhibitors, the nuclei and cytosol of the guard cells exhibited a higher level of oxidation (about 10%) than the other cell types, but they were much less susceptible to high levels of inhibitor-mediated oxidation. Moreover, the oxidation degree in the cytosol was significantly less changed by the LINC and ANT treatments than the oxidation degree in the nuclei, perhaps suggesting that the presence of chloroplasts (even if bleached) serves to protect against inhibitor-mediated oxidation.

The data presented here show that NF, LINC, ANT and SHAM decreased the abundance of the *LHCA1* and *LHCB1* transcripts. Inhibition of mitochondrial electron transport therefore has a negative impact on PhANG expression. Like ANT, NF and LINC significantly increased *AOXA1* expression, suggesting that inhibition of chloroplast electron transport has a positive effect on NGEMP expression. Although each inhibitor may lead to the production of different forms of ROS in different cell types, exposure to all the inhibitors led to oxidative stress in all the cell types studied. Under the conditions used in the present experiments, the expression of *LHCA1*, *LHCB1* and *AOXA1* was significantly decreased, although the extent of repression of *AOXA1* was much less marked than that of the nuclear-encoded photosynthesis genes. The expression of AOX1 is highly responsive to environmental stress [30], being highly expressed in conditions that require alleviation of electron pressure within the respiratory electron transport chain [30,31]. However, few studies have measured the extent of oxidation imposed during the stress treatments that induce AOX1 expression. The expression of nuclear genes such as *LEA5* and *RRFT1* was generally enhanced by the oxidative stress imposed by all the inhibitors. While the RRFT1 transcription factor is a nuclear protein, LEA5 protein is localized in mitochondria [32,33]. Interestingly, the levels of *WHIRLY1* and *WHIRLY3* transcripts, which encode single-stranded DNA binding proteins found in either in chloroplasts (WHIRLY3) alone or chloroplasts and the nucleus (WHIRLY1), were also significantly increased by the inhibitors, except for the NF plus LINC combination, which resulted in mRNA levels similar to the controls [33].

Although the addition of chloroplast or mitochondrial inhibitors represents an artificial system that is far removed from the physiological conditions experienced by the plant, such treatments have been widely used to identify PhANGs and NGEMPs [16,21]. The involvement of redox signalling in chloroplast-to-nucleus signalling pathways has previously been considered in NF-treated seedlings, and estimated using transcript markers [8]. Lower levels of NF (5 nM) have been used together with lower irradiances of NF-treated seedlings in an attempt to circumvent severe oxidative stress [34]. This led to the identification of the *happy on norflurazon* (*hon*) mutants, which remained green in the presence of NF [34]. The *HON* genes include ClpR4 (*HON5*), a nucleus-encoded subunit of the chloroplast-localized Clp protease complex, and a putative chloroplast translation elongation factor (*HON23*). However, a marker gene for hydrogen peroxide-induced stress responses, *FERRETIN1*, was upregulated in *hon5* and *hon23* mutants even in the absence of the inhibitor, suggesting that these mutants were pre-acclimated to enhanced oxidation. More studies are clearly required that simultaneously measure gene expression and cellular redox status. This study, which involved 5 days exposure to 5 µM NF, shows that under these conditions, the roots and cotyledons of the seedlings experience severe oxidative stress. These data suggest that this screen therefore detects signalling pathways that reflect a general oxidative stress response. Further work using ro-GFP expressing seedlings exposed to the different concentrations of inhibitors for shorter times is required to fully analyse the role of oxidative signalling circuits in organelle to nucleus signalling.

Multiple pathways are likely to be involved in the anterograde and retrograde signalling that controls the expression of PhANGs and NGEMPs. It is currently difficult to determine the extent to which such pathways are integrated through the redox signalling hub. For example, HSP90 proteins, which are ubiquitous chaperones that are important in plant stress responses, have a role in tetrapyrrole-mediated control of PhANG expression that is dependent on the HY5 transcription factor [7]. Moreover, the ABI4 transcription factor, which mediates redox signalling, is also important in chloroplast-to-nucleus and mitochondria-to-nucleus signalling [17,18,21,22]. PhANG expression was less decreased following LINC or NF treatment in the *abi4* mutant than the wild-type [15,30]. It is likely multiple transcription factors including NAC-type transcription factors such as ANAC017, and WRKY transcription factors (e.g. WRKY15 and WRKY40) participate in the coordination of retrograde signalling pathways. For example, a recent study has provided evidence that ANAC017 and *flu*-mediated singlet oxygen signalling arising from different pathways may converge on multiple common target genes [30].

In conclusion, these data show that the presence of inhibitors such as LINC or ANT that impair chloroplast or mitochondrial functions lead to coordinated expression of nuclear genes targeted to both organelles. Oxidative processing thus provides a nexus of coordination for signals passing between the chloroplasts, mitochondria and nuclei, and not just between the chloroplasts and nucleus or the mitochondria and nucleus alone. The cellular redox signalling hub may therefore be seen as a key integrator of retrograde signals arising from different organelles, allowing communication between different cellular compartments and the nucleus.

## Data accessibility

This article has no additional data.
